# Myositis

**DOI:** 10.1007/s00393-022-01278-2

**Published:** 2022-10-13

**Authors:** Peter Korsten, Eugen Feist

**Affiliations:** 1 Klinik für Nephrologie und Rheumatologie, Universitätsmedizin Göttingen, Robert-Koch-Str. 40, 37075 Göttingen, Deutschland; 2 Klinik für Rheumatologie und Klinische Immunologie, Helios Fachklinik Vogelsang-Gommern, Sophie-von-Boetticher-Str. 1, 39245 Vogelsang-Gommern, Deutschland

**Keywords:** Myositis, Leitlinie, Immunsuppressive Therapie, Interstitielle Lungenerkrankung, Myositis, Guidelines, Immunosuppressive therapy, Interstitial lung disease

## Abstract

Unter der Federführung der Deutschen Gesellschaft für Neurologie und Beteiligung vieler weiterer Fachgesellschaften wurde die S2k-Leitlinie zu Myositissyndromen vollständig aktualisiert und überarbeitet. Bei der Klassifikation der Myositiden werden nun die immunmediierte nekrotisierende Myopathie und das Antisynthetasesyndrom als eigenständige Entitäten aufgefasst. Bezüglich der Diagnostik gibt die Leitlinie konkrete Empfehlungen zum Dysphagiescreening, insbesondere bei der Einschlusskörperchenmyositis, und zur Tumordiagnostik bei bestimmten Myositisformen. In der Therapie steht nach der positiven ProDERM-Studie mit intravenösen Immunglobulinen (Octagam®) ein zugelassenes Präparat zur Verfügung. Auf Basis der INBUILD-Studie ist Nintedanib als antifibrotische Therapie bei progressiv fibrosierender Lungenbeteiligung verfügbar. Die aktualisierte Leitlinie stellt ein für Rheumatolog*innen praxistaugliches Dokument mit zahlreichen Empfehlungen zur Versorgung von Myositispatient*innen dar.

## Abstract (en)

The S2e guidelines on myositis were completely updated and revised under the leadership of the German Society for Neurology and the participation of many other specialist societies. Immune-mediated necrotizing myopathy and antisynthetase syndrome are now regarded as independent entities in the classification of myositis. With respect to the diagnostics, the guidelines provide concrete recommendations on dysphagia screening, especially for inclusion body myositis and for cancer diagnostics in certain forms of myositis. Following the positive ProDERM study, the use of intravenous immunoglobulins (Octagam®) is available for treatment as an approved substance. Based on the INBUILD study, antifibrotic treatment with nintedanib is available for progressive fibrosing pulmonary involvement. For rheumatologists, the updated guidelines represent a document relevant for daily practice with many recommendations for the treatment of patients with myositis.

Zu Myositissyndromen sind zwischenzeitlich wichtige neue Erkenntnisse gewonnen worden, weshalb Expert*innen für diese Erkrankungen unter Federführung der Deutschen Gesellschaft für Neurologie eine aktualisierte S2k-Leitlinie erarbeitet haben, deren wichtigste neue Inhalte wir hier vorstellen [2].

## Neues zur Klassifikation

Eine wichtige Neuerung in der Klassifikation stellt die Einführung der immunvermittelten nekrotisierenden Myopathie (IMNM) als eigenständige Entität dar, die klinisch schlecht von der Polymyositis (PM) zu unterscheiden ist und als spezifische Marker Antikörper (AK) gegen „signal recognition particle“ (SRP) oder 3‑Hydroxy-3-Methylglutaryl-CoA-Reduktase (HMGCR) aufweist. Ebenso wird das Antisynthetasesyndrom (ASyS, synonym verwendet ASS) mit spezifischen AK (am häufigsten: Anti-Jo1) nun als von anderen Myositiden abzugrenzende Form aufgefasst. Somit gliedern sich die idiopathischen inflammatorischen Myositiden nunmehr in die Polymyositis (PM), IMNM, Dermatomyositis (DM), Overlap-Myositis (OM), ASyS und die Einschlusskörperchenmyositis „(inclusion body myositis“ [IBM]).

## Besonderheiten in der Diagnostik

Auf klinisch wichtige Besonderheiten wie die starke Assoziation zu Tumorerkrankungen bei DM mit Anti-TIF1-γ-AK oder die mögliche rapid-progressive interstitielle Lungenerkrankung („interstitial lung disease“ [ILD]) bei Anti-MDA5-AK-Nachweis weisen die Autor*innen der Leitlinie explizit hin.

Weiterhin findet sich nun auch erstmalig ein eigenes Kapitel zur Checkpoint-Inhibitor-assoziierten Myositis.

Neben der etablierten diagnostischen Aufarbeitung (klinische Untersuchung inklusive standardisierter Muskelkraftmessung) sollen die Bestimmung Myositis-spezifischer und -assoziierter Antikörper sowie eine Muskelbiopsie erfolgen. Dabei sollte die Muskelbiopsie detailliert immunhistologisch und ggf. elektronenmikroskopisch aufgearbeitet werden.

Insbesondere bei der IBM sollte zudem explizit nach Schluckstörungen gefragt werden. Eine spezielle HNO-ärztliche und ggf. endoskopische Schluckuntersuchung (Videofluoroskoptest) ist dann indiziert. In der Behandlung dieser Manifestation bei IBM ergab z. B. die Therapie mit einer lokalen Botulinumtoxin-Injektion zum Teil gute Ergebnisse (aktuelle Übersicht bei [5]).

In der Initialdiagnostik und im Verlauf werden Besonderheiten der Myositisformen dargestellt und auch konkrete Empfehlungen zur Tumordiagnostik bei erhöhtem Risiko gegeben, die aufgrund ihrer Bedeutung hier wörtlich zitiert werden:

Erhebung der Anamnese inklusive B‑Symptomatik, ausführliche körperliche Untersuchung inklusive Hautbefund, CT-Thorax und -Abdomen (alternativ: Röntgen-Thorax, Sonographie des Abdomens) sowie gynäkologische, urologische und gastroenterologische Screeninguntersuchung bei allen Myositis-Patienten zur Diagnosestellung. Für Patienten mit dem Nachweis von Anti-TIF1‑γ, Anti-NXP‑2 und Anti-HMGCR-Antikörpern wird die Durchführung einer FDG-PET-CT oder alternativ einer Ganzkörper-CT bei Diagnosestellung empfohlen. Eine Wiederholung dieser Screening-Maßnahmen sollte innerhalb des ersten Jahres nach Diagnosestellung erfolgen und in Abhängigkeit von individuellen Risikofaktoren erneut, auch mehrfach, im Zeitraum der folgenden zwei Jahre. Für alle Myositis-Patienten sollten hiernach entsprechend Alter und Risikofaktoren die üblichen Krebs-Vorsorgeuntersuchungen erfolgen. [2]

## Neue zugelassene Therapieoptionen: Immunglobuline (Octagam®) bei Dermatomyositis und Nintedanib bei chronisch progressiver Lungenbeteiligung

Hinsichtlich der immunsuppressiven/immunmodulierenden Behandlungskonzepte ist für die tägliche Praxis relevant, dass Rituximab als therapeutische Alternative („off-label“) insbesondere bei therapieresistenten Fällen sowohl bei der DM als auch bei der PM Erwähnung findet. Weiterhin wurden die kürzlich publizierten Ergebnisse der kontrollierten *ProDERM*-Studie mit i.v. Immunglobulinen (IVIg) bei DM hervorgehoben: Im Vergleich zu Placebo hatten Patient*innen mit einer IVIg-Therapie nach 16 Wochen signifikant positive Effekte auf einen kombinierten Endpunkt [1]. Darauf folgte international die Zulassung für IVIg (Octagam®, Octapharma AG, Lachen, Schweiz) für die Behandlung der DM. Hier ist darauf hinzuweisen, dass der Gemeinsame Bundesausschuss mit Beschluss von April 2013 eine Off-label-Behandlung mit Immunglobulinpräparaten bei PM und DM stützt (abrufbar unter: https://www.g-ba.de/downloads/39-261-1701/2013-04-18_AM-RL-VI_OLU_IVIG-PM-DM_BAnz.pdf).

Für die (progressiv fibrosierende) ILD als eine der schwersten Komplikationen von Myositiden ist auf Basis der INBUILD-Studie eine zugelassene antifibrotische Therapie mit Nintedanib (Ofev®, Boehringer-Ingelheim, Ingelheim am Rhein, Deutschland) verfügbar, die in der Leitlinie mit starkem Konsens empfohlen wird [3]. Bei rapid-progressiven ILD-Formen (RP-ILD), die insbesondere bei der oft klinisch amyopathischen, anti-MDA5-AK-positiven Dermatomyositis („clinically amyopathic dermatomyositis“ [CADM]) vorkommen kann, ist eine aggressive Kombinationstherapie erforderlich. In refraktären Fällen kommt auch eine Plasmapherese in Betracht [4].

## Besondere Situationen: Impfen, Schwangerschaft und Stillzeit

Die aktuelle Leitlinie enthält nun auch explizite Empfehlungen zu juvenilen Myositisformen und zur Therapie in besonderen Situationen wie Schwangerschaft und Stillzeit. Weiterhin geben die Autor*innen der Leitlinie Hinweise zu Impfungen, die sich am von der Ständigen Impfkommission empfohlenen Vorgehen orientieren.

## Bedeutung der Heilmittelversorgung

Neben Konsensusempfehlungen zur Diagnostik und medikamentösen Therapie herrscht zwischen den Beteiligten Expert*innen aus Neurologie, Rheumatologie und Dermatologie sowie Patientenvertreter*innen eine große Einigkeit darüber, dass ergänzende Heilmittel (Ergotherapie, Logopädie, Physiotherapie, Rehabilitation) ebenfalls eine wichtige Rolle in der Behandlung von Myositissyndromen spielen.

Zusammenfassend stellt die aktualisierte und überarbeitete Leitlinie zu Myositissyndromen ein praxistaugliches und wichtiges Dokument dar, das viele klinisch relevante Aspekte umfasst.

## Fazit für die Praxis

Die aktualisierte Fassung der S2k-Leitlinie zu Myositissyndromen gibt zahlreiche konkrete und praxisrelevante Empfehlungen zur Diagnostik und Therapie von Myositiden.

## Interessenkonflikt

P. Korsten gibt unabhängig von diesem Artikel Reisekostenunterstützung von AbbVie und Chugai an und erhielt Vortrags- und Beratungshonorare von AbbVie, Amgen, AstraZeneca, Boehringer Ingelheim, Bristol-Myers-Squibb, Chugai, Galapagos, GlaxoSmithKline, Janssen-Cilag, Lilly, Pfizer und Sanofi-Aventis. Zudem erhielt seine Institution Studiendrittmittel von AstraZeneca, Boehringer-Ingelheim, Diamed Medizintechnik GmbH, Galapagos, GlaxoSmithKline und Novartis, ebenfalls nicht in Verbindung mit diesem Artikel. P. Korsten ist aktuell zweiter Vorsitzender der *International Myositis Society*. E. Feist gibt unabhängig von diesem Artikel Berater- und Gutachtertätigkeiten für AbbVie, BMS, Lilly, MSD, Roche, Novartis, Pfizer und Sanofi-Aventis, Mitarbeit in „advisory boards“ von BMS, Lilly, MSD, Novartis und Sanofi-Aventis sowie Vortragshonorare von Lilly und Novartis an. Zudem erhielt er Studiendrittmittel von Novartis, ebenfalls unabhängig von diesem Artikel. Beide Autoren haben als Vertreter der Deutschen Gesellschaft für Rheumatologie bei der Erstellung der Leitlinie mitgewirkt.
